# Metabolic comorbidities and the association with risks of recurrent metastatic disease in breast cancer survivors

**DOI:** 10.1186/s12885-021-08343-0

**Published:** 2021-05-22

**Authors:** Sumadi Lukman Anwar, Roby Cahyono, Dayat Prabowo, Widya Surya Avanti, Lina Choridah, Ery Kus Dwianingsih, Wirsma Arif Harahap, Teguh Aryandono

**Affiliations:** 1 Division of Surgical Oncology - Department of Surgery, Dr. Sardjito Hospital / Faculty of Medicine, Public Health, and Nursing, Universitas Gadjah Mada, Jl. Kesehatan No. 1, Yogyakarta, 55281 Indonesia; 2 Department of Radiology, Dr. Sardjito Hospital/Faculty of Medicine, Public Health, and Nursing, Universitas Gadjah Mada, Yogyakarta, 55281 Indonesia; 3 Department of Anatomical Pathology, Dr. Sardjito Hospital / Faculty of Medicine, Public Health, and Nursing, Universitas Gadjah Mada, Yogyakarta, 55281 Indonesia; 4 Division of Surgical Oncology, Dr. M Jamil Hospital / Faculty of Medicine, Universitas Andalas, Padang, 25127 Indonesia

**Keywords:** Obesity, Metabolic, Diabetes, Dyslipidemia, Breast cancer

## Abstract

**Background:**

Obesity and other metabolic comorbidities affect over 10% of patients with breast cancer and are closely related with adverse outcomes. Although metabolic comorbidities among breast cancer patients in low- and middle-income countries are suggested to be lower, only a few studies are currently available. Effective management of metabolic comorbidities in cancer patients has been associated with better outcomes.

**Methods:**

Non-metastatic breast cancer patients (*N* = 1081) treated in our department (2014–2018) were monitored for the presence of high Body Mass Index (BMI), diabetes or glucose intolerance, dyslipidemia, and hypertension and the development of recurrent metastatic diseases during a median follow-up of 3.9 years.

**Results:**

Glucose intolerance, hypertension, dyslipidemia, and BMI ≥ 27.7 kg/m^2^ considered at risk for metabolic comorbidities were found in 26.5, 42.6, 27.7, and 23.3% of breast cancer patients, respectively. Diabetes or glucose intolerance and having both glucose intolerance and dyslipidemia were associated with the risk of recurrent metastatic disease (OR = 1.442, 95%CI = 1.071–1.943, *p* = 0.016 and OR = 1.495, 95%CI = 1.090–2.049, *p* = 0.010; respectively). Having three or more metabolic comorbidities was significantly associated with the risk of recurrent metastatic disease (OR = 1.647, 95%CI = 1.139–2.382, *p* = 0.008) compared to patients without any comorbidity. The metabolic comorbidities were distributed unevenly among breast cancer subtypes. A significant association with recurrent metastatic disease was found in the Luminal B-like subtype. In post-menopausal patients, having more than three comorbidities was associated with a higher risk of recurrent metastatic disease compared to those without any comorbidity (OR = 2.000, 95%CI = 1.035–3.067, *p* = 0.001). The risks of having three or more metabolic comorbidities were significantly higher in breast cancer survivors who were obese, lived in an urban area, and received hormonal therapy of aromatase inhibitors.

**Conclusion:**

Metabolic comorbidities were frequently found in breast cancer patients and were associated with higher risks to develop recurrent metastatic disease, particularly in post-menopausal women. Subsequent larger studies are needed to better understand the association of metabolic comorbidities with patients’ quality of life and prognosis, and to explore the potential combination of clinical intervention and lifestyle modification in breast cancer survivors to treat as well as reduce their impact.

**Supplementary Information:**

The online version contains supplementary material available at 10.1186/s12885-021-08343-0.

## Background

Breast cancer incidence has been continuously increasing, making it a major public health problem among women worldwide [1]. Over 2 million women are diagnosed with breast cancer every year, and it is projected to increase by 30% in 2035 [1, 2]. The outcome of breast cancer has improved tremendously due to the advances of comprehensive treatment involving multidisciplinary teams as well as the implementation of early detection and breast cancer screening [3]. Although the breast cancer incidence in low- and middle-income countries (LMICs) is lower, the ratio of mortality-to-incidence is 3.4 times higher than in high-income countries [3]. In solid tumor carcinoma including breast cancer, the vast majority of cancer-related deaths are caused by distant metastasis [4], although the current advancements of breast cancer treatment can prolong the patients’ lifespan and help to improve their quality of life.

Excess weight and other metabolic-related disorders are increasing in every part of the world including LMICs [5, 6]. Widespread adaptation into high-calorie diet and sedentary lifestyle are among the most hypothesized contributors to the global rise of overweight and dyslipidemia [5]. In addition, populations in LMICs often suffer from a double burden of obesity and undernutrition, in which Indonesia prevails as the most severely affected country with this problem [7]. In association with cancer, chronic metabolic-related disorders are associated with a significantly elevated risk of breast cancer [8, 9]. Additionally, the presence of metabolic comorbidities in patients with breast cancer has also been closely related to adverse outcomes [10, 11].

The vast majority of breast cancer survivors are over 50 years old and are in a post-menopausal period [12]. Therefore, comorbidities related to aging such as adiposity, glucose intolerance, dyslipidemia, and hypertension are higher in breast cancer survivors than the general population [12, 13]. Breast cancer treatment particularly chemotherapy and hormonal therapy might also divert endocrine function thus predisposing patients to a higher risk of metabolic disorders [14, 15]. Having chronic disorders is often not efficiently managed in patients with a primary diagnosis of cancer [16], and the condition might be worse in the areas of the world with limited health resources. Modification of lifestyle through physical activity, dietary programs, as well as alcohol and tobacco cessation and effective clinical management are very important to treat metabolic comorbidities and might also be able to improve breast cancer patients’ prognosis and quality of life [17].

Although the association of metabolic comorbidities and the unfavorable impacts on breast cancer prognosis is largely known, most studies derive their data from high-income countries. The evidence is mostly developed from the patients in the early stages of breast cancer at diagnosis with a much longer period of overall survival and in the settings where clinical guidelines and health systems are already well-established [18]. Information about frequency and relative impacts of metabolic comorbidities on breast cancer progression from LMICs including Indonesia are very limited. Identification and effective management of metabolic comorbidities are also challenging particularly in settings with limited health resources. This study estimated individual components and concomitant metabolic comorbidities and their association with breast cancer progression in overall breast cancer patients as well as in stratified groups according to intrinsic breast cancer subtypes and menopausal status.

## Material and methods

### Study participants

Demographic, clinical, and pathological data of non-metastatic breast cancer patients who underwent surgery in 2014–2018 (*N* = 1081) at Dr. Sardjito Hospital were analyzed to predict the potential association between metabolic comorbidities and risk of recurrent metastatic breast cancer. Dr. Sardjito Hospital is a referral unit treating almost 60% of patients with breast cancer living in Yogyakarta and the southern part of the Central Java provinces. All female patients with definitive diagnosis of breast cancer who received standard treatment and at least 6 months of follow-up at the Department of Surgery, Dr. Sardjito Hospital were recruited. Follow-up was performed according to the institutional guidelines for breast cancer survivors. This retrospective cohort study was conducted following the ethical standards adopted in the 1964 Declaration of Helsinki. The study was approved by the Medical and Health Research Ethics Committee Faculty of Medicine, Public Health, and Nursing - Universitas Gadjah Mada Yogyakarta (1049/EC/2018).

### Data mining

Basic demographic data (age, education, residence), clinical (cancer stages, surgery, and administrated treatment of chemotherapy, hormonal therapy, and radiotherapy) and pathological (tumor size, lymph node involvement, histological grades, vascular and perineural infiltration) characteristics were collected from the individual patient’s medical charts. Tumor-Node-Metastasis (TNM) system of the 7th Edition of the American Joint Committee on Cancer (AJCC) [19] was used to determine breast cancer stages. Guidelines from the World Health Organization (WHO) [20] were used to classify cancer histological types. Histological cancer grade was stratified according to the modified Bloom and Richardson system (mSBR) [21]. Breast cancer subtypes were grouped according to modified criteria of the St. Gallen Consensus 2013 [22, 23] using a positive or negative expression of estrogen receptor (ER), progesterone receptor (PR), human epidermal growth factor receptor 2 (HER2), and Ki-67 as previously reported [22, 23].

Breastfeeding was classified as yes if maintained for at least 1 year. Residence was grouped into urban or rural area according to legal information from the patient’s identity card shown during the first hospital visit. Parity was classified according to the number of full-term pregnancies. Education levels were binarily classified into lower than high school and high school or university/college. Menarche was determined as age at first menstruation and was then classified into early menarche (≤ 12 years), normal menarche (13–14 years), and late menarche (≥ 15 years). Menopause was defined as the age at which menstruation ceased.

### Metabolic comorbidities

Metabolic risk parameters including body mass index (BMI), blood pressure, and glucose levels were screened at baseline. Measurements of weight, height, and blood pressure were performed by nurses during the hospital visit and documented in the medical records. BMI was then calculated as weight (kg) divided by height in meters squared. BMI ≥ 27.7 kg/m^2^ as a substitute for waist circumference ≥ 88 cm was used as a cutoff for metabolic comorbidity and was subsequently defined as BMI at risk [18]. Hypertension was defined as either systolic ≥140 mmHg or diastolic ≥90 mmHg evaluated in ≥2 blood pressure measurements [24]. Dyslipidemia at baseline was tested if any other metabolic comorbidity was found at diagnosis. Dyslipidemia that was detected before or after chemotherapy was included to determine presence of metabolic comorbidities. Dyslipidemia was defined as blood high-density lipoprotein (HDL) value < 50 mg/dL or low-density lipoprotein (LDL) value > 150 mg/dL or total cholesterol level > 200 mg/dL, or triglyceride level > 150 mg/dL. Diabetes or impaired glucose intolerance was defined as fasting glucose level ≥ 100 mg/dL or postprandial glucose level ≥ 140 mg/dL or HbA1c level ≥ 6.5% in ≥2 measurements [25].

### Recurrent metastatic breast cancer

In this study, recurrent metastatic breast cancer was characterized as the presence of any distant organ metastasis during follow-up and surveillance. Metastasis was defined as disease manifestation in the distant organ particularly the lungs, bone, liver, and brain showed by clinical evidence and confirmed with imaging/pathology examination or radiologic changes using computed tomography imaging with contrast or whole-body bone scan. Disease progression was defined as the presence of new tumor growth or relapse or cancer spread after the period of complete treatment. Monitoring of recurrent disease was performed according to the local institutional guidelines. Follow-up visits at least once a month were generally scheduled during the first 6 months after acute treatment of surgery, chemotherapy, and radiotherapy. Subsequent regular visits for clinical examination and imaging evaluation were arranged every 6 months unless any unscheduled visit was indicated. During each surveillance visit, a thorough clinical examination and imaging evaluation including breast-axillae ultrasonography and/or mammography, abdominal sonography, thoracic X-ray, and bone scan were evaluated. Mammography was performed every 2 years. However, if a patient had any complaints indicating locoregional and distant metastasis, further examination would be performed in addition to the scheduled routine imaging. Any finding of recurrent metastatic disease was documented until the last date of follow-up in September 2019.

### Statistical analysis

Baseline characteristics were presented in frequency tables to compare attributable demographic and clinicopathological variables among patients with or without metabolic comorbidities. Continuous variables among subgroups were compared using the Mann-Whitney-U tests and categorical variables were compared using the χ2 tests. The association was further analyzed with multivariable logistic regression using recurrent metastatic breast cancer during follow-up as a dependent variable and individual or concomitant metabolic comorbidities as covariates. The association was mainly presented as odds ratio (OR) accompanied by 95% confidence intervals (CI) and *p* < 0.05 was defined as statistically significant value. All statistical tests were completed using SPSS 17.0 software (IBM Corp., Chicago).

## Results

### Demography and frequency of metabolic comorbidities in breast cancer survivors

Baseline demography and clinicopathological characteristics of 1081 patients with non-metastatic breast cancer are summarized in Table 1. Means of age at diagnosis and for BMI were 50.9 years old and 24.08 kg/m^2^. The majority of patients were diagnosed after menopause (71.7%, *N* = 775) and mean of age at menopause was 47 years old. Around 98% (*N* = 1059) were Javanese origin and resided in the rural region (71%, *N* = 768). Around 80% of patients were diagnosed in Stage III breast cancer with mean of tumor size of 6.3 cm and 62.2% (*N* = 780) were positive axillary lymph node (Table 1).

**Table 1:** Baseline characteristics of breast cancer patients (stage I-III) at diagnosis and the distribution between those with and without any metabolic comorbidity

Variables	Category	Overall	With any metabolic comorbidity	Without any metabolic comorbidity	*P* value
Age (years)		1081 (100%)	606 (56.1%)	475 (43.9%)	
	Mean (SD)	50.94 (10.5)	52.5 (10.1)	49.0 (10.7)	< 0.001
	≤35	77 (7.1%)	58 (7.1%)	50 (10.5%)	
	36–40	110 (10.2%)	102 (12.4%)	64 (13.5%)	
	41–55	526 (48.7%)	381 (46.5%)	226 (47.6%)	
	56–65	277 (25.6%)	201 (24.5%)	106 (22.3%)	
	> 65	91 (8.4%)	78 (9.5%)	39 (6.1%)	
Ethnicity	Javanese	1059 (98.0%)	598 (98.7%)	461 (97.1%)	0.06
	Non-Javanese	22 (2.0%)	8 (1.3%)	14 (2.9%)	
Residence	Rural	768 (71%)	426 (70.3%)	342 (72%)	0.540
	Urban	313 (29%)	180 (29.7%)	133 (28%)	
Menarche (years)	≤12	190 (17.6%)	112 (18.5%)	78 (16.4%)	0.377
	13–14	514 (47.5%)	305 (50.3%)	209 (44.0%)	
	≥15	377 (34.9%)	189 (31.2%)	188 (39.6%)	
Menopausal status	No (pre-menopause)	306 (28.3%)	137 (22.6%)	169 (35.6%)	0.001
	Yes (post-menopause)	775 (71.7%)	469 (77.4%)	306 (64.4%)	
Age at menopause	≤50	593 (54.9%)	354 (58.4%)	239 (50.3%)	0.033
	> 50	182 (16.8%)	115 (19.0%)	67 (14.1%)	
Parity	Nulliparous	120 (11.1%)	58 (9.6%)	62 (13.1%)	0.071
	Multiparous	961 (88.9%)	548 (90.4%)	413 (86.9%)	
Breastfeeding	No	216 (20.0%)	114 (18.8%)	102 (21.5%)	0.277
	Yes	865 (80.0%)	492 (81.2%)	373 (78.5%)	
BMI	≤18.5	136 (12.6%)	49 (8.1%)	87 (18.3%)	< 0.001
	18.6–25	534 (49.4%)	217 (35.8%)	317 (66.7%)	
	25.1–30	302 (27.9%)	231 (38.1%)	71 (14.9%)	
	> 30	109 (10.1%)	109 (18.0%)	0 (0%)	
Family history	Yes	199 (18.4%)	99 (16.3%)	100 (21.1%)	0.057
	No	882 (81.6%)	507 (83.7%)	375 (78.9%)	
Histology grade	I	7 (0.6%)	1 (0.6%)	6 (1.2%)	0.058
	II	210 (19.4%)	128 (21.1%)	82 (17.3%)	
	III	864 (79.9%)	477 (78.7%)	387 (81.5%)	
Stage	I	13 (1.2%)	5 (0.8%)	8 (1.7%)	0.061
	II	356 (32.9%)	217 (35.8%)	139 (29.3%)	
	III	712 (65.9%)	384 (63.4%)	328 (69.1%)	
Tumor size	≤ 2 cm	51 (4.7%)	26 (4.3%)	25 (5.3%)	0.038
	2–5 cm	311 (28.8%)	193 (31.8%)	118 (24.8%)	
	> 5 cm	719 (66.5%)	387 (63.9%)	332 (69.9%)	
Node status	N0	301 (27.8%)	181 (29.9%)	120 (25.3%)	0.220
	N1	547 (50.6%)	306 (50.5%)	241 (50.7%)	
	N2	191 (17.7%)	97 (16.0%)	94 (19.8%)	
	N3	42 (3.9%)	22 (3.6%)	20 (4.2%)	
Adjuvant endocrine therapy	No	462 (42.7%)	234 (38.6%)	228 (48.0%)	0.001
	Tamoxifen	319 (29.5%)	176 (29.0%)	143 (30.1%)	
	Aromatase inhibitor	300 (27.8%)	196 (32.3%)	104 (21.9%)	
Subtype	Luminal-B (*N* = 497)	497 (46.0%)	297 (59.8%)	200 (40.2%)	0.02
	Luminal-B (*N* = 123)	123 (11.4%)	78 (63.4%)	45 (36.6%)	
	Her2- enriched (*N* = 174)	174 (16.1%)	95 (54.6%)	79 (45.4%)	
	TNBC (*N* = 287)	287 (26.5%)	135 (47.0%)	152 (53.0%)	
(Neo-)/Adjuvant chemotherapy	No	92 (8.5%)	39 (6.4%)	53 (11.2%)	0.006
	Yes	989 (91.5%)	567 (93.6%)	422 (88.8%)	
Adjuvant radiotherapy	No	285 (26.4%)	157 (25.9%)	128 (26.9%)	0.700
	Yes	796 (73.6%)	449 (74.1%)	347 (73.1%)	
Surgery	Mastectomy	975 (90.2%)	540 (89.1%)	435 (91.6%)	0.351
	BCT	99 (9.2%)	61 (10.1%)	38 (8.0%)	
	Biopsy	7 (0.6%)	5 (0.8%)	2 (0.4%)	
Metabolic comorbidities	DM or glucose intolerance	287 (26.5%)	287 (26.5%)	0 (0%)	–
	Dyslipidemia	299 (27.7%)	299 (27.7%)	0 (0%)	
	Hypertension	464 (42.9%)	464 (42.9%)	0 (0%)	
	BMI risk (> 27.7)	252 (23.3%)	252 (23.3%)	0 (0%)	

In this study,  606 of 1081 (56.1%) patients with breast cancer had at least one metabolic comorbidity. Among the metabolic comorbidities, hypertension was the most frequently diagnosed (42.9%), followed by dyslipidemia, diabetes or glucose intolerance, and weight risk, with 287 (26.5%), 299 (27.7%), and 252 (23.3%), respectively (Table 1).

### Presence of metabolic comorbidities and the association with risk of recurrent metastatic disease and locoregional progression

After diagnosis and receiving treatment of breast cancer, patients were monitored for the presence of recurrent distant metastasis as an outcome in this study. Table 2 summarizes the period of follow-up in which 281 of 1081 (26%) patients progressed into distant organ metastasis. Univariable Cox regression analysis showed that diabetes or impaired glucose intolerance was significantly associated with risk of recurrent metastatic disease (OR = 1.442, 95%CI: 1.071–1.943, *p* = 0.016) and the risks were higher in the co-occurrence of glucose intolerance with dyslipidemia (OR = 1.495, 95%CI: 1.090–2.049, *p* = 0.013) and with hypertension (OR = 1.626, 95%CI: 1.122–2.356, *p* = 0.010, Table 3). Having three or more metabolic comorbidities was significantly associated with distant metastasis (OR = 1.622, 95%CI: 1.822–2.226, *p* = 0.003). In comparison to patients with no metabolic comorbidities, those with three or more metabolic comorbidities were at risk of distant organ metastasis (OR = 1.647, 95%CI: 1.139–2.382, *p* = 0.008). Multivariable regression of metabolic comorbidities was significantly able to predict recurrent metastatic disease among breast cancer patients with F(12,1068) = 2.664, *p* = 0.002 in which having more than three comorbidities had 27.4% efficiency, *p* = 0.001. When locoregional breast cancer recurrences were included in the analysis, however, the metabolic comorbidities were not significantly associated with risks of disease progression (Table 4).

**Table 2:** Period of breast cancer follow-up after treatment to monitor the presence of recurrent metastatic diseases

Follow-up (month)	N (%)
6–12	2 (0.2%)
13–24	135 (12.5%)
25–36	198 (18.3%)
37–48	277 (25.6%)
49–60	155 (14.3%)
> 61	314 (29.0%)

**Table 3:** Odds ratios and 95% confidence intervals for association individual and concurrent metabolic comorbidities and risks of recurrent metastatic disease using binominal logistic regression, *N* = 1081. Individual and concurrent metabolic variables were included in the multivariable analysis

Presence of metabolic comorbidities	With distant metastasis event N (%)	Without distant metastasis event N (%)	OR	95%CI	*P* value
DM or glucose intolerance	90 (32%)	197 (24.6%)	1.442	1.071–1.943	**0.016**
Dyslipidemia	78 (27.7%)	221 (27.6%)	1.007	0.746–1.364	0.966
Hypertension	118 (41.9%)	346 (43.25%)	0.950	0.721–1.251	0.714
BMI risk	66 (23.5%)	186 (23.2%)	1.013	0.735–1.397	0.935
DM/glucose intolerance + Dyslipidemia	51 (18.1%)	96 (12.0%)	1.626	1.122–2.356	**0.010**
DM/glucose intolerance + Hypertension	76 (27.0%)	159 (19.9%)	1.495	1.090–2.049	**0.013**
Dyslipidemia + Hypertension	75 (26.7%)	204 (25.5%)	1.064	0.781–1.448	0.695
Dyslipidemia + BMI risk	32 (11.4%)	93 (11.6%)	0.977	0.638–1.497	0.915
Hypertension + BMI risk	45 (16.0%)	125 (15.6%)	1.030	0.710–1.493	0.877
DM/glucose intolerance + BMI risk	23 (8.2%)	59 (7.4%)	1.120	0.678–1.850	0.678
≥3 metabolic comorbidities	77 (27.4%)	151 (18.9%)	1.622	1.182–2.226	**0.003**
Any metabolic comorbidity	146 (53.6%)	460 (56.7%)	0.875	0.664–1.152	0.341

**Table 4:** Odds ratios and 95% confidence intervals for association individual and concurrent metabolic comorbidities and risk of locoregional progression using binominal logistic regression, *N* = 1081. Individual and concurrent metabolic variables were included in the multivariable analysis

Presence of metabolic comorbidities	With locoregional progression N (%)	Without locoregional progression N (%)	OR	95%CI	*P* value
DM or glucose intolerance	149 (25.2%)	138 (28.2%)	0.853	0.650–1.118	0.249
Dyslipidemia	153 (25.8%)	146 (29.7%)	0.786	0.524–1.132	0.172
Hypertension	237 (41.9%)	225 (45.9%)	0.850	0.671–1.161	0.213
BMI risk	132 (22.3%)	120 (24.6%)	0.886	0.682–1.201	0.386
DM/glucose intolerance + Dyslipidemia	76 (12.8%)	71 (14.4%)	0.886	0.625–1.256	0.496
DM/glucose intolerance + Hypertension	123 (20.8%)	112 (22.8%)	0.860	0.643–1.151	0.311
Dyslipidemia + Hypertension	145 (24.5%)	134 (27.3%)	0.789	0.614–1.142	0.296
Dyslipidemia + BMI risk	62 (10.5%)	63 (12.8%)	0.771	0.621–1.201	0.252
Hypertension + BMI risk	86 (14.5%)	85 (17.3%)	0.803	0.661–1.193	0.241
DM/glucose intolerance + BMI risk	40 (6.8%)	42 (8.5%)	0.775	0.494–1.216	0.267
≥3 metabolic comorbidities	118 (20.0%)	110 (22.4%)	0.821	0.698–1.194	0.331
Any metabolic comorbidity	317 (53.6%)	289 (58.9%)	0.701	0.552–1.098	0.085

### Metabolic comorbidities among different intrinsic breast cancer subtypes

Patients with breast cancer were intrinsically grouped according to the expression of ER, PR, Her2, and Ki67 [22, 23] into Luminal A-like (*N* = 497), Luminal B-like (*N* = 497), Her2 enriched (*N* = 497), and TNBC subtypes (*N* = 497). Among these subtypes, only Luminal B-like showed a significant association  of glucose intolerance OR = 5.697, 95%CI: 2.278–14.248, *p* = 0.001), concurrent glucose intolerance with dyslipidemia OR = 9.100, 95%CI: 2.495–33.196, *p* = 0.001), concurrent glucose intolerance with hypertension OR = 5.467, 95%CI: 2.124–14.072, *p* < 0.001), and more than three metabolic comorbidities with recurrent metastatic disease (OR = 5.014, 95%CI: 1.969–12.767, *p* = 0.001) (Table 5). Binary subclassification into Luminal (*N* = 620) and Non-Luminal (*N* = 461) showed significant association only in Luminal subtypes, i.e., concurrent glucose intolerance and dyslipidemia and having more than three metabolic comorbidities with a risk of recurrent metastatic disease (OR = 1.655, 95%CI: 1.038–2.683, *p* = 0.034 and OR = 1.705, 95%CI: 1.143–2.543, *p* = 0.009; respectively), Supplementary Table 1. Multivariable regression in the Luminal B-like subtype of metabolic comorbidities significantly predicted recurrent metastatic disease among breast cancer patients with F(12,110) = 2.905, *p* = 0.002 in which having more than three comorbidities had 76.5% efficiency, *p* = 0.001.

**Table 5:** Odds ratios and 95% confidence intervals for the association of metabolic comorbidities across different breast cancer subtype with the risk of recurrent metastatic disease using binary logistic regression. Individual and concurrent metabolic variables were included in the multivariable analysis

Variables	Luminal A (*N* = 497)			Luminal B (*N* = 123)			Her2-enriched (*N* = 174)			TNBC (*N* = 287)		
	OR	95%CI	*P* value	OR	95%CI	*P* value	OR	95%CI	*P* value	OR	95%CI	*P* value
DM or glucose intolerance	1.054	0.680–1.632	0.815	5.697	2.278–14.248	**< 0.001**	2.004	0.967–4.149	0.061	1.022	0.434–2.405	0.961
Dyslipidemia	0.837	0.543–1.291	0.422	1.548	0.644–3.723	0.329	1.068	0.483–2.360	0.871	1.204	0.635–2.282	0.569
Hypertension	0.782	0.522–1.172	0.234	1.791	0.759–4.229	0.183	1.111	0.561–2.202	0.763	0.972	0.563–1.679	0.919
BMI risk (> 27.7)	1.145	0.730–1.798	0.514	1.410	0.544–3.653	0.480	0.730	0.293–1.820	0.499	0.795	0.416–1.520	0.488
DM/glucose intolerance + Dyslipidemia	1.227	0.732–2.058	0.438	9.100	2.495–33.196	**0.001**	1.513	0.569–4.026	0.407	1.675	0.758–3.705	0.202
DM/glucose intolerance + Hypertension	1.069	0.670–1.704	0.781	5.467	2.124–14.072	**< 0.001**	2.304	1.071–4.959	**0.033**	1.187	0.618–2.281	0.607
Dyslipidemia + Hypertension	0.893	0.613–1.378	0.613	1.812	0.748–4.391	0.188	1.167	0.525–2.593	0.705	1.150	0.600–2.206	0.672
Dyslipidemia + BMI risk	1.002	0.568–1.766	0.995	0.916	0.237–3.543	0.899	1.245	0.308–5.034	0.759	0.855	0.350–2.089	0.730
Hypertension + BMI risk	1.001	0.598–1.677	0.997	1.258	0.410–3.860	0.688	1.027	0.348–3.032	0.962	1.000	0.474–2.109	1.000
DM/glucose intolerance + BMI risk	1.076	0.549–2.108	0.831	2.359	0.374–14.874	0.361	0.811	0.162–4.053	0.798	1.099	0.410–2.942	0.852
≥3 metabolic comorbidities	1.330	0.850–2.082	0.212	5.014	1.969–12.767	**0.001**	1.447	0.602–3.482	0.409	1.576	0.817–3.040	0.175
Any metabolic comorbidity	0.833	0.556–1.247	0.374	2.554	0.948–6.879	0.064	0.905	0.458–1.788	0.773	0.794	0.469–1.344	0.391

### Metabolic comorbidities in pre- and post-menopausal breast cancer

As the majority of patients were diagnosed after menopause (71.7%, *N* = 775) and any metabolic comorbidity was higher in post-menopausal women, we performed univariate logistic regression of each metabolic component as shown in Table 6. In post-menopausal breast cancer patients, glucose intolerance, concurrent glucose intolerance with dyslipidemia, concurrent glucose intolerance with hypertension, and having more than three metabolic comorbidities were associated with risk of recurrent metastatic disease (OR = 1.569, 95%CI: 1.110–2.217, *p* = 0.011 and OR = 1.714, 95%CI: 1.134–2.591, *p* = 0.011, OR = 1.705, 95%CI: 1.191–2.441, *p* = 0.004 and OR = 1.967, 95%CI: 1.377–2.810, *p* < 0.001; respectively), Table 6. Multivariable regression of metabolic comorbidities in post-menopausal women also significantly predicted recurrent metastatic disease with only more than three comorbidities having contribution of 25% efficiency, *p* = 0.001.

**Table 6:** Odds ratios and 95% confidence intervals showing the association of metabolic comorbidities and the risk of recurrent metastatic disease in pre- (*N* = 306) and post-menopausal breast cancer patients (*N* = 775) using binominal logistic regression. All variables were included in the multivariable analysis

Variables	Pre-menopause (*N* = 306)			Post-menopause (*N* = 775)		
	OR	95%CI	*P* value	OR	95%CI	*P* value
DM or glucose intolerance	1.425	0.760–2.671	0.269	1.569	1.110–2.217	**0.011**
Dyslipidemia	0.816	0.426–1.565	0.541	1.142	0.805–1.621	0.456
Hypertension	0.686	0.395–1.191	0.181	1.151	0.828–1.600	0.401
BMI risk (> 27.7)	0.629	0.314–1.263	0.193	1.229	0.851–1.775	0.271
DM/glucose intolerance + Dyslipidemia	1.935	0.774–4.839	0.546	1.714	1.134–2.591	**0.011**
DM/glucose intolerance + Hypertension	1.257	0.610–2.592	0.535	1.705	1.191–2.441	**0.004**
Dyslipidemia + Hypertension	0.785	0.395–1.559	0.899	1.240	0.871–1.765	0.233
Dyslipidemia + BMI risk	0.602	0.164–2.211	0.445	1.133	0.715–1.793	0.595
Hypertension + BMI risk	0.335	0.097–1.161	0.084	1.317	0.881–1.970	0.180
DM/glucose intolerance + BMI risk	0.274	0.034–2.224	0.226	1.404	0.827–2.384	0.209
≥3 metabolic comorbidities	1.083	0.489–2.399	0.845	1.967	1.377–2.810	**< 0.001**
Any metabolic comorbidity	0.727	0.444–1.191	0.206	1.088	0.775–1.526	0.626

### Increased risks of distant metastasis with an accumulation number of metabolic comorbidities

In comparison to breast cancer survivors without any metabolic comorbidity, the risk to develop recurrent metastatic disease was significantly higher particularly in the presence of two comorbidities (OR 1.751, 95%CI: 1.137–2.695, *p* = 0.011) and three or more metabolic comorbidities (OR 1.647, 95%CI: 1.139–2.382, *p* = 0.008). The risks were higher for having three or more metabolic comorbidities in Luminal B-like subtypes and post-menopausal women (OR 6.500, 95%CI: 2.090–20.216, *p* = 0.001 and OR 2.000, 95%CI: 1.035–3.067, *p* = 0.001; respectively), Table 7. In addition, having glucose intolerance and hypertension was associated with higher risks of recurrent metastatic disease in Her-2 enriched subtype (OR 2.304, 95%CI: 1.071–4.959, *p* = 0.033), Table 5.

**Table 7:** Odds ratios and 95% confidence intervals for association of number of metabolic comorbidities with risk of recurrent metastatic disease in overall patients, Luminal B-like subtypes, and post-menopausal women

Number of metabolic comorbidities	Overall (*N* = 1081)			Luminal B (*N* = 123)			Post menopause (*N* = 775)		
	OR	95%CI	*P* value	OR	95%CI	*P* value	OR	95%CI	*P* value
0	ref	ref		ref	ref	ref	ref	ref	
1	0.795	0.537–1.177	0.252	1.806	0.486–6.703	0.377	0.923	0.566–1.506	0.748
2	1.751	1.137–2.695	**0.011**	1.230	0.281–5.376	0.783	1.712	1.003–2.924	**0.049**
3+	1.647	1.139–2.382	**0.008**	6.500	2.090–20.216	**0.001**	2.000	1.035–3.067	**0.001**

### Variables associated with the development of multiple metabolic comorbidities in patients with breast cancer

We further analyzed determinants predisposing patients to have multiple metabolic comorbidities. Univariable regression analysis showed that age more than 40 years, living in an urban area, menarche younger than 14 years old, breastfeeding practice, the preexistence of obesity, and receiving adjuvant hormonal therapy particularly aromatase inhibitors were associated with an increased risk of developing multiple metabolic comorbidities (Table 8). Multivariable regression analysis found that only the preexistence of obesity, older age, and receiving hormonal therapy significantly contributed to the development of multiple metabolic comorbidities in patients with breast cancer (*R*^2^ = 0.318, *p* < 0.001).

**Table 8:** Odds ratios and 95% confidence intervals for the association of demographic and socioeconomic determinants with risk of having 3 or more metabolic comorbidities among breast cancer survivors. All variables were included in the multivariable analysis

Variables	References	Association		
		OR	95%CI	*P* value
Age				
> 40 years	< 40 years	3.322	1.946–5.673	**< 0.001**
Ethnicity				
Javanese	Non-Javanese	1.208	0.404–3.597	0.736
Residence				
Urban	Rural	1.550	1.138–2.112	**0.005**
Age at menarche				
< 14 years	> 14 years	1.408	1.024–1.936	**0.035**
Parity				
Multiparity	Nulliparity	1.477	0.884–2.469	0.136
Breastfeeding				
Yes	No	1.548	1.038–2.308	**0.032**
Marital status				
Married	Not married	0.691	0.388–1.232	0.211
Education				
Lower than high school	High school or higher	0.754	0.558–1.019	0.066
BMI				
≥30	< 30	5.120	3.386–7.742	**< 0.001**
Family history				
Yes	No	0.964	0.659–1.410	0.852
Stage at diagnosis				
I-II	III	1.373	1.016–1.855	**0.039**
Neo−/Adjuvant Chemotherapy				
Yes	No	1.296	0.740–2.269	0.364
Adjuvant hormonal therapy				
Yes	No	1.500	1.108–2.033	**0.009**
Aromatase inhibitor	Tamoxifen	2.416	1.647–3.546	**< 0.001**
Adjuvant radiotherapy				
Yes	No	0.781	0.569–1.078	0.133

## Discussion

Obesity, impaired glucose intolerance, dyslipidemia, and elevated blood pressure are the closely interrelated conditions associated with not only an increased risk of breast cancer but also with an adverse prognosis of breast cancer survivors [8, 9, 11]. Most studies reporting the association of metabolic comorbidities with breast cancer prognosis arise from early-stage breast cancer in the high income countries [11, 18]. Since the frequency of obesity and other metabolic disorders differ among regions and socioeconomic levels [6], the impact on patients with breast cancer might also be different. In our cohort consisting of breast cancer survivors who were mostly diagnosed in late stages and from low socioeconomic status, metabolic comorbidity was found in more than 50% of patients. Using binary logistic regression, glucose intolerance, dual comorbidity of glucose intolerance and dyslipidemia, and more than three comorbidities were associated with a significant risk to develop recurrent metastatic disease, especially among patients with Luminal B-like subtype and among postmenopausal women. Our findings are in concordance with previous studies showing the association of metabolic comorbidities with poor prognosis of breast cancer [11, 18]. Berrino et al. [26] specifically proposed that having three or more metabolic comorbidities predisposed patients with breast cancer for significant risk of distant metastasis (OR 2.45). Glucose intolerance has also been associated with higher disease recurrence and shorter overall survival in patients with breast cancer [27].

Having more than three metabolic comorbidities was consistently associated with increased risks to develop recurrent metastatic disease compared to breast cancer patients without having metabolic comorbidities (Table 3). The risks were higher in Luminal B-like subtype and post-menopausal women (Table 6). However, metabolic comorbidities were not significantly associated with elevated risks of breast cancer locoregional recurrences (Table 4). Association of metabolic comorbidities in post-menopausal breast cancer patients with poor prognosis showed mixed results [28, 29]. Parallel with Zhang et al. [30] and Dibaba et al. [11], we identified adverse effects of metabolic comorbidities with higher risk of having recurrent metastatic disease in post-menopausal breast cancer patients. Although we found a significant association of glucose intolerance, and in combination with dyslipidemia and hypertension, as well as having three or more metabolic comorbidities in the Luminal B-like subtype with the risk of recurrent metastatic disease, previous studies found a significant association of metabolic comorbidities with TNBC-subtype [31, 32]. Patterns of hormonal-related factors have been distinctly reported among Asian patients with breast cancers compared to Caucasian including significantly lower proportion of postmenopausal patients at diagnosis and hormonal receptor positivity [33]. Insulin resistance has been reported in 26.4% of patients with breast cancers and is significantly associated with Luminal-B subtype among Asian patients [34]. Obesity, visceral adiposity, and insulin resistance have been postulated to stimulate breast carcinogenesis by activating proto-oncogenes and antiapoptotic transcriptional factors partially through ER-related pathways [35, 36]. Significant association of having two metabolic comorbidities with recurrent metastatic disease in Her2-enriched breast cancer (OR 2.304, Table 5) might also need further study because the preexisting comorbidities will potentially increase cardiotoxicity events [37].

Metabolic comorbidities predispose patients with breast cancer for distant metastasis through several biological pathways [17]. Weight risk and abdominal adiposity are suggested to induce the conversion of androgen into estrogen through aromatase activation [17, 38]. Estrogen is a key player in breast carcinogenesis through the promotion of cell division and inhibition of cell survival [38]. Hormonal switch after menopause also causes a striking alteration in lipid and carbohydrate metabolism thus predisposing women to dyslipidemia, central adiposity, and ageing [39]. In addition, central adiposity has also been associated with an increased risk of impaired glucose intolerance, hypertension, and dyslipidemia. Insulin resistance causes hyperinsulinemia, a condition that induces mitogenic signaling pathways including cell proliferation, angiogenesis, and cell migration [40]. Therefore, obesity and insulin resistance are also associated with disease progression and worse prognosis in patients with breast cancer [40]. In addition, underlying chronic inflammation in individuals with weight risk, glucose intolerance, and dyslipidemia is also suggested to have a high risk of breast cancer progression [41]. Chronic inflammation accelerates cancer metastasis through reorganization of the tumor environment and intrinsic tumor cell properties that fertilize both the primary tumor and the soil of systemic tissues to enhance extravasation, inoculation, and growth of vasculature [42, 43].

We further identified risk factors for the development of multiple metabolic comorbidities among breast cancer survivors (Table 8). Prerequisite obesity, older age, and living in an urban area were significantly associated with a higher risk of having three or more metabolic comorbidities indicating a socioeconomic gradient that needs to be addressed. Previous studies showed that inequalities in socioeconomic backgrounds such as occupation, levels of education, and gender were associated with risks of metabolic disease clustering [44, 45]. In accordance with previous reports [14, 15], adjuvant endocrine treatment particularly aromatase inhibitors also predisposed patients to higher risks of metabolic comorbidities indicating the need for careful consideration and prevention for patients at higher risks [14, 15, 46]. Adjuvant hormonal treatment has been associated with significant survival improvement of patients with ER-positive breast cancer [47], although subsequent adverse risk in the lipid profile, bone health, cardiovascular events are continuously being evaluated to mitigate the risks [48]. Our study also showed that early stage breast cancers had significantly higher risk of developing multiple metabolic comorbidities (OR 1.373, Table 7) probably due to longer follow-up and survivorship than late stage cancers as also shown by other studies [49, 50]. Adjuvant cancer treatments have been associated with adverse metabolic parameters that also affect options to receive subsequent treatment with significant gradients according to the cancer stages [46, 49, 50].

Our present study underscores the need to effectively manage metabolic comorbidities following successful acute treatment of breast cancer to further prevent recurrent metastatic disease. In the settings where health resources are limited, clinicians have faced significant challenges to meet the high standard breast cancer care [51] as well as to manage the presence of metabolic comorbidities. Even in high-income countries, metabolic comorbidities in breast cancer are often not optimally managed [52]. Several causal factors are low patient compliance and medical accessibility [52]. In LMICs, the figures might even be worse not only because of limited health resources but also low insurance ownership, lack of awareness, and complicated referral systems [3, 53, 54]. One of the most cost-effective interventions both to prevent and to manage metabolic comorbidities is a lifestyle change. However, more than a third of cancer survivors could not engage in regular exercise and weight management [55]. Among other factors, one of the most striking is the lack of advice and guidance from their oncologists [56]. Schmitz et al. [55] as well as the American Cancer Society [57] have proposed guidelines for clinicians on how to implement physical exercise programs for patients with cancer. Our further challenges include the utilization of limited resources [54] to deliver lifestyle interventions to prevent metabolic diseases as well as provide a thorough clinical treatment to efficiently manage metabolic comorbidities as part of the continuum of breast cancer treatment to prevent disease recurrence and progression [58].

Several strengths of our present study were the development of additional evidence in the different setting of a breast cancer cohort of individual and concomitant metabolic comorbidity with an increased risk of distant metastasis, and the particular association with breast cancer subtypes i.e., Luminal B-like, and post-menopausal women. The association of metabolic comorbidities with patient’s social-economic gradients might also provide additional information for local public health policymakers to design a cost-effective program in reducing chronic metabolic diseases at both clinical and community levels. The limitations of this study existed in the nature of retrospective studies including the involvement of other risk factors for cancer progression and small sample size, particularly in the subgroup analysis. Dyslipidemia was not routinely screened, and several metabolic risks were estimated using surrogate measurements i.e., visceral adiposity with BMI > 27.7. In addition, although metabolic comorbidities play a role in breast cancer progression, other factors might also contribute to the recurrent metastatic disease including skin and chest wall infiltration, positive axillary lymph nodes, tumor size, Her2 expression, and intrinsic biological profiles of the primary tumor [59, 60]. Risks of locoregional and distant metastasis recurrences also largely depend on the residual cancer statuses (R0, R1, and R2) after surgical resection that are not specifically addressed in this study. Cox proportional hazard-model was not used because the variables of metabolic comorbidities were not constant which might contribute to the study’s limitations. In addition, premature loss of follow-up and censoring due to withdrawal could influence the interpretations which might be limited as shown in the Table 2. Larger and multisite studies with longer follow-up are needed to extend and confirm our findings. In addition, an evaluation of lifestyle interventions, clinical management, patient adherence, and development of a model of an efficient referral system in the treatment of metabolic comorbidities among breast cancer survivors also constitute very important considerations in the field of oncology.

## Conclusions

We identified individual and coexisting metabolic comorbidities with a risk of breast cancer progression defined as recurrent distant metastasis. In addition, we found relative socioeconomic gradients with the frequency of metabolic comorbidities among breast cancer patients that might reflect the prerequisite of lifestyle and increased levels of health awareness which need to be further addressed. Improving guidelines and developing an efficient clinical management system for metabolic comorbidities in patients with breast cancer are also needed to help patients achieve the targeted weight, glucose, and blood lipid levels and to reduce the potential risk of developing recurrent metastatic disease.

## Supplementary Information

**Additional file 1: Supplementary Table 1.** Odds ratios and 95% confidence intervals showing the association of metabolic comorbidities with the risk of distant metastasis in Luminal and Non-Luminal breast cancer subtypes. All variables were included in the multivariable analysis.

## Data Availability

The dataset is available upon reasonable request to the corresponding author.

## Abbreviations

AJCC: American Joint Committee on Cancer
BMI: Body mass index
BSA: Body Surface Area
DM: Diabetes mellitus
EC: Ethical Clearance
ER: Estrogen receptor
HDL: High density lipoprotein
Her2: Human epidermal growth factor receptor-2
LMIC: Low- and middle-income countries
LDL: Low density lipoprotein
mSBR: Modified Bloom and Richardson system
OR: Odds ratio
PR: Progesterone receptor
SD: Standard deviation
SE: Standard Error
TG: Triglycerides
TNM: Tumor node metastasis
TNBC: Triple negative breast cancer
WHO: World Health Organization
