# Supplementary material for: Metabolic comorbidities and the association with risks of recurrent metastatic disease in breast cancer survivors
Source: BMC Cancer. 2021 May 22;21:590. doi: 10.1186/s12885-021-08343-0 (PMC8141199; doi:10.1186/s12885-021-08343-0)
Supplement: Supplementary file 1 — Additional file 1: Supplementary Table 1. Odds ratios and 95% confidence intervals showing the association of metabolic comorbidities with the risk of distant metastasis in Luminal and Non-Luminal breast cancer subtypes. All variables were included in the multivariable analysis. [file 12885_2021_8343_MOESM1_ESM.docx]

Supplementary Table 1. Odds ratios and 95% confidence intervals showing the association of metabolic comorbidities with the risk of distant metastasis in Luminal and Non-Luminal breast cancer subtypes. All variables were included in the multivariable analysis.

| Variables | Luminal (N=620) | | | Non-Luminal (N=461) | | |
| --- | --- | --- | --- | --- | --- | --- |
|  | OR | 95%CI | p value | OR | 95%CI | p value |
|  |  |  |  |  |  |  |
| ­DM or glucose intolerance | 1.445 | 0.980-2.131 | 0.063 | 1.449 | 0.910-2.307 | 0.119 |
| Dyslipidemia | 0.942 | 0.639-1.387 | 0.761 | 1.145 | 0.696-1.882 | 0.594 |
| Hypertension | 0.907 | 0.632-1.303 | 0.234 | 1.020 | 0.667-1.561 | 0.927 |
| BMI risk (>27.7) | 1.192 | 0.793-1.792 | 0.398 | 0.774 | 0.457-1.313 | 0.343 |
| DM/glucose intolerance + Dyslipidemia | 1.655 | 1.038-2.638 | **0.034** | 1.605 | 0.867-2.971 | 0.132 |
| DM/glucose intolerance + Hypertension | 1.462 | 0.969-2.205 | 0.070 | 1.553 | 0.948-2.541 | 0.080 |
| Dyslipidemia + Hypertension | 1.023 | 0.690-1.515 | 0.690 | 1.154 | 0.697-1.910 | 0.578 |
| Dyslipidemia + BMI risk | 0.996 | 0.591-1.679 | 0.988 | 0.954 | 0.451-2.022 | 0.903 |
| Hypertension + BMI risk | 1.047 | 0.655-1.672 | 0.997 | 1.012 | 0.548-1.896 | 0.970 |
| DM/glucose intolerance + BMI risk | 1.194 | 0.636-2.239 | 0.581 | 2.359 | 0.374-14.874 | 0.361 |
| ≥3 metabolic comorbidities | 1.705 | 1.143-2.543 | **0.009** | 1.529 | 0.904-2.587 | 0.113 |
| Any metabolic comorbidity | 0.994 | 0.688-1.437 | 0.374 | 0.832 | 0.549-1.259 | 0.383 |
|  |  |  |  |  |  |  |
